# Influence of Reproductive Status: Home Range Size in Water Voles (*Arvicola amphibius*)

**DOI:** 10.1371/journal.pone.0154338

**Published:** 2016-04-26

**Authors:** Karl Frafjord

**Affiliations:** Department of Natural Sciences, Tromsø University Museum, UiT The Arctic University of Norway, Tromsø, Norway; University of Missouri Kansas City, UNITED STATES

## Abstract

The relationship between home range and reproductive status of water voles (*Arvicola amphibius*) was studied by radio-tracking on an island off the coast of northern Norway in 2006–2009. The aim was to test assumptions about the species’ social structure relative to other microtines. Juveniles used fairly small ranges (about 400 m²), with no difference between males and females. Subadults, overwintered voles in April, had ranges similar to juveniles. Reproductively active males (mean 2774.0 m²) increased their range seven-fold relative to juvenile males, with ranges on average 3.3 times larger than adult females (mean 848.3 m²), which also expanded their range. Most litters were born in May and June, and as reproduction ceased in July adult males reduced their range whilst females did not. Body mass or year did not influence home range size. Overlap of home ranges varied, but could be extensive in both adult males and females. The water vole had a social structure similar to some *Microtus* species, but females appeared to be non-territorial and males perhaps conditioned territorial and non-territorial.

## Introduction

Home range, the area utilized by an animal, is an important biological parameter related to sex, age, body size, activity, diet, reproduction, survival and predator avoidance [1, 2]. Spacing behaviour and mating systems are important parts of a species’ social organization, often with characteristic sex specific differences in home range size. The distribution of resources, evenly or patchily, is an important determinant of spacing behaviour and territoriality (the exclusive use of space), and the costs and benefits of resource defence often vary with time.

Social organization has been studied in detail for some microtines, see [3, 4]. In the genus *Microtus*, large inter- and intraspecific variations in social organization have been found, whereas *Myodes* voles appear to have a more fixed organization [5–11]. Most species are thought to be territorial, at least during the reproductive season, either through individual or group defence of an area and groups are in most cases formed by close kin [12–15]. Groups based on kinship may be common in the largely non-breeding winter season, but most break up at the onset of reproduction in spring, although male bank voles (*Myodes glareolus*) appear to continue to share a common home range during the summer [16–18]. In microtines, cooperative defence of resources or raising young seem to be almost absent, except in some monogamous species where males care for young. Food preferences (grasses, foliage or omnivory) are important determinants for social organization, with species that largely eat grasses being less territorial than others.

In small mammals, males often are polygynous, with ranges overlapping several adult females [19]. Territorial defence in females is mostly related to food resources, shelter or nest sites, but exclusive territories may be needed to counter infanticide, with other females posing a higher risk than males [20]. Optimal foraging theory predicts that females should defend a territory with enough resources both for herself and her offspring, either seasonally or yearly. Polygynous males on the other hand, should maximize the number of receptive females in their range during the breeding season, when larger males could be expected to use larger ranges than smaller males, but may in other seasons optimize habitat resources for survival. In some mammals, young form dominance hierarchies (often by sex), with the most dominant individuals having the first choice in selecting a place to live, *sensu* [21]. Subordinate young may “sit and wait”, choose to leave or be forcibly repelled, the outcome possibly being density-dependent. Mortality, which is regularly very high in small mammals, may potentially influence social organization both on a spatial and temporal scale, by reducing density and freeing up space.

The social organization of water voles *Arvicola* spp. is not particularly well studied [22]. Most studies have been done in the United Kingdom, were the water vole (*A*. *amphibius*) often live along streams and rivers giving very narrow ranges [23–25]. Under these circumstances females are territorial with small, non-overlapping breeding ranges, whereas male ranges are larger (longer), often overlapping, related to body mass and at least partly determined by social factors [26–29]. The more terrestrial form of the species living on grasslands is less well studied in this respect. One largely unpublished study from Sweden [30, 31] found differences related to habitat, i.e. males had much larger home ranges in marshland than in grassland. In marshland, males had larger ranges than females, whereas in grassland they did not, but they behaved apparently territorial in both habitats and increased density lead to decreased range size. During the non-breeding season all water voles were solitary and territorial [31].

Understanding the mechanisms influencing social organization of voles living under different conditions is basic to understanding their population biology and conservation. Which type of social structure is found in the northern part of the water vole’s distribution range? One of the main objectives of this study was to consider effects of reproductive status on home range size in the terrestrial form of the water vole, another to consider effects of social interactions. I examined the following predictions; 1) adult water voles have significantly larger home ranges than juveniles, 2) males of both age groups have larger home ranges than females, 3) range size reflects reproductive status and will change with season (from pre-breeding to breeding to post-breeding), 4) range size in adults varies with year (possibly resulting from variations in population density), and 5) male-male home ranges overlap relatively less than female-female home ranges (males are more territorial). The results indicate that reproduction *per se* has a major impact on the home range of water voles.

## Material and Methods

### Ethics statement

Trapping and marking water voles with ear tags and radio collars were authorized by the Norwegian Directorate for Nature Management (now The Norwegian Environment Agency). Because anaesthetization was not needed the animals could be released immediately at the site of capture. I attempted to remove all radio collars from animals at the end of a study period or when a transmitter failed, and succeeded in all but two cases. I considered this marking as simple identity tagging, which does not require special permit (according to § 2 in the Norwegian legislation “Forskrift om forsøk med dyr”, an unauthorised translation to English can be found in: http://oslovet.norecopa.no/statute.html). No animals were injured or sacrificed and handling took only a few minutes, reducing the handling stress as much as possible. Furthermore, I only collared the minimum number of animals required. The land owners gave permission to work on their property (all islands in the archipelago are privately owned), which was not protected. The water vole in Norway is classified as a species of “least concern” and is not endangered.

### Study site

Water voles were studied on a small island in the archipelago Solvær in Nordland (UTM 33W 0388 7357), northern Norway, just south of the Arctic Circle. The vegetation on this tree-less island was generally heath interspersed with some grassland and boggy areas, and most voles lived in grassland. The water vole is the only rodent on the island and the only other consumer of vegetation was greylag geese (*Anser anser*) in summer. The main predator of voles is the eagle owl (*Bubo bubo*) [32], but other birds of prey were also seen on the island at various times. In most years only one eagle owl was present in the territory and no breeding was recorded during the study period. The mink (*Neovison vison*) does not live in this archipelago. Water voles have been studied since 2003, with every captured vole being marked with individually numbered ear tags (type #1005–1, National Band and Tag Co., USA), and sexed, aged and weighed using a spring scale (Pesola 300 or 600 g, 5 and 10 g graduation) with the vole held in a cotton bag. Radio-collared voles were weighed when fitting and removing the collar.

Generally, I used 36 custom-made live traps [33] placed at entrances or in runways, moving them successively over the study (trapping) area over a period of 6–8 days. Note that the trapped area was smaller than the total area used by radio collared voles and that trapping periods did not equal tracking sessions. In 2007–2008, I modified the normal trapping scheme of two short periods per year (spring and late summer) to intermittent trapping when disturbance to radio tracked individuals could be minimized. Reproduction took place from April to July, with nearly all litters born in May and June. Among 720 juveniles caught in 2006–2009, only one was born in July (based on extrapolating a growth curve) and none later. The single one caught indicated a litter with more pups.

### Radio-tracking

Water voles were fitted with radio collars (PIP2, Biotrack Ltd., UK, mass 3 g) in 2006–2009. Voles were collared as they were caught, in the first or second day of each tracking session in which collars were fitted. Handling did not require anaesthesia and voles were released immediately at the site of capture. A minimum body mass limit of ca. 140 g was set for collaring juveniles (radio collar = 2.1% of body mass), with only one exemption (see results). The transmitter had a theoretical life-length of 3 months, but rarely lasted more than 2 months. In most cases the antennae broke off after 1–2 months, which made tracking more difficult and entailed more frequent homing-in on the animal to verify its position. Normally, I kept a distance of minimum 5 m to the vole, mostly walking along regular paths. Many voles habituated to my presence, enabling a number of observations. The receiver (FM-100, Advanced Telemetry Systems, Inc., USA) was used with handheld Yagi antennae (Televilt International AB, Sweden). By collaring only voles trapped within a limited part of the study area, i.e. in a relatively small area, I was able to study their interactions in terms of overlapping ranges. The total study area was about 15000 m² (1/10^th^ of the island).

**Fig 1:**
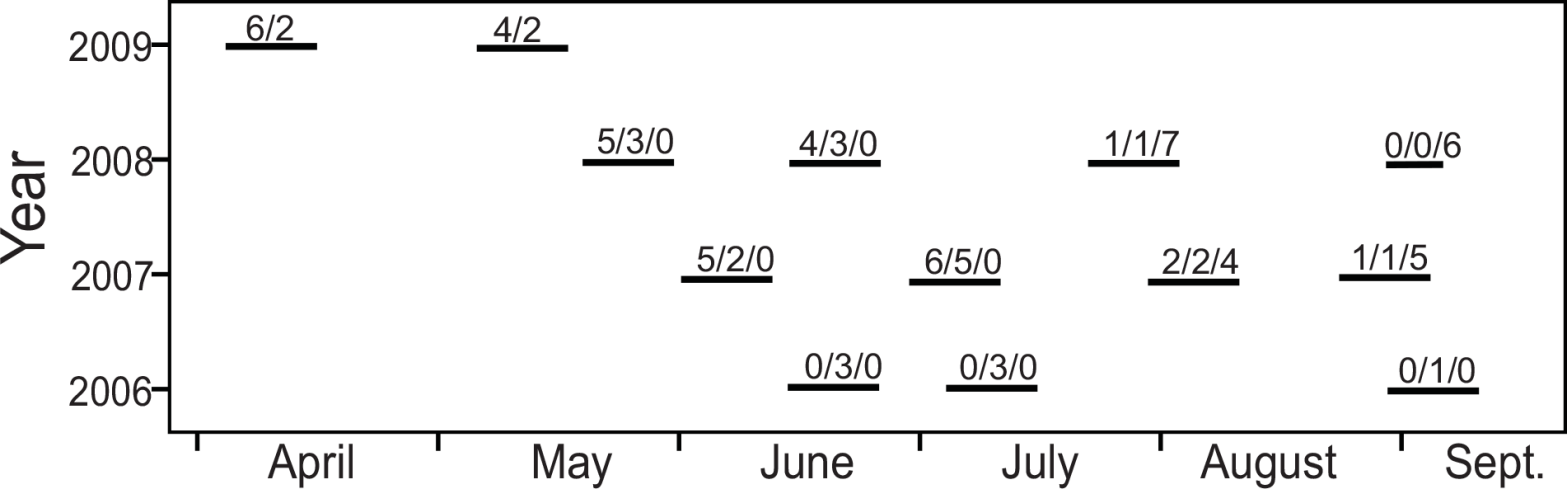
Schematic illustration of tracking sessions (lines) between April and September in four years. Numbers refer to the number of water voles studied in each session: adult males/adult females/juveniles (in April 2009: subadult males/subadult females).

The length and timing of tracking sessions are given in Fig 1. Radio-collared water voles were tracked for on average 21.3±8.8 days (minimum 9, maximum 51 days, n = 44 voles) and in 2.0±0.7 sessions (maximum 4 sessions for transmitters with maximum longevity). A session lasted on average 11.6 days, when all collared voles were tracked daily for 10–15 hours. Voles were tracked mostly in daytime hours (08.00–24.00), but all 24 hours over several days were covered at least once. Recordings (fixes) were made every 15 minutes in 2006 (only 3 animals) and every 30 minutes in other years, giving minimum 20 fixes per animal per day (regularly 25–35 fixes per animal per day, more in 2006), and a minimum of 270 fixes in the first session. Running at high speed, a water vole was able to cross the entire length of even the largest range in 5–10 minutes. The tracking scheme reduced autocorrelation between fixes and maximized the number of fixes per day. Positions were recorded using a handheld GPS (GPSmap 60Cx, Garmin, Inc., USA) by triangulation, homing in on the animal if needed, but as a rule no closer than 5 meters. Positions were recorded to the nearest meter, although the positioning error given by the GPS was ±2–4 m. I was often able to see the vole and thus verify its exact position. All voles spent a particular large amount of time in certain areas, termed “nesting”(or “core“) areas, artificially defined as 5x5 m wide. Having established the approximate position of that area, I did not normally enter it, leaving the voles undisturbed a large part of the time. This implies that in most cases when the animal was inside its “core” area the exact location was not further pinpointed.

Particular attention was given to fixes outside the hitherto known range of the animal. I often waited until the animal had left before approaching the spot to read the accurate position. Trapping positions of collared individuals were also included in the data set. At the end of the study, collars were removed from all animals still alive, except two females in 2006 that did not enter any trap. Both of these were observed in September at close range with apparently no ill effects of the collar, but they were not retrapped the next spring (overall, the number of voles that survived two winters was nearly nil, in 2006–2009 the number was zero). All collars from dead animals and all lost collars were retrieved.

### Data analyses

Adults were defined as overwintered and reproductively active voles, identified by their fur (they keep the long, shaggy winter fur throughout most of the summer), size and body mass. Juveniles were defined as young of the year, only two juveniles were suspected to be pregnant in their first summer during 2006–2009, but their litters were not verified. Subadults were defined as overwintered animals in April only. Overall, 45 voles were radio collared, of which 43 were used in the analysis. A total of 11 adult males, 14 adult females, 7 juvenile males and 5 juvenile females were radio collared from May to August. In addition, 6 male and 2 female subadults were collared in April 2009, to study the transition to adult range.

One adult female lost her collar underground during the first day and was not included in any statistical analysis. For one adult male I subtracted the area of a small bay from his home range size. He travelled between both sides of the bay, but not across water and including sea water would have overestimated his range. For another adult male I dismissed one fix, the last trapping site when the malfunctioning transmitter was removed, because it was so utterly outside his previous range (*ca*. 20 m) and would have increased his range disproportionately much (he was unwilling to enter any trap inside his regular home range in this late period and may have left it due to my trapping efforts). This was the only outlier that was excluded, *i*.*e*. the only “excursion” outside of a home range. In April, one male used a disproportionately small home range, and was not included in home range analyses because it seemed abnormal, although he seemed to be healthy and may not have been sick (more information in the results). Two other males were predated between these two tracking sessions and are included only as subadults. The other 5 subadult voles from 2009 are included both as juveniles in April and adults in May (sample size was considered more important and worth the small risk of violating the statistical test with repeated measures).

I estimated home range size by the 100% minimum convex polygon method (MCP, software Tracker ver. 1.1, Radio Location Systems AB, Sweden), both per tracking session and overall. MCP only utilizes relatively few fixes along the margin of ranges, but gave very reasonable configurations and sizes of all home ranges. This method may slightly overestimate a range compared to the actual range, but this does not affect its power in comparing ranges. The overall individual MCP is the combined MCP estimated from several sessions, except for one vole that used completely different ranges during two sessions (as subadult and adult in 2009) and these were summed to avoid overestimating its range (*i*.*e*., the sum was smaller than overall MCP). “Tracker” was also used to estimate zones of overlap between dyadic ranges (excluding non-overlapping ranges). Percentage overlap of ranges was calculated from the mean home range size for the demographic group. Overall home range size was used in these analyses.

Data are presented as mean±1 SD. Statistical methods include Generalized Linear Model (GLMZ), Spearman’s rank correlation, t-test, Mann-Whitney U test, Wilcoxon signed ranks test (z), and Chi-square test of independence (IBM SPSS Statistics). Both the range in various sessions and the overall range across all sessions are used as units of analysis.

## Results

**Fig 2:**
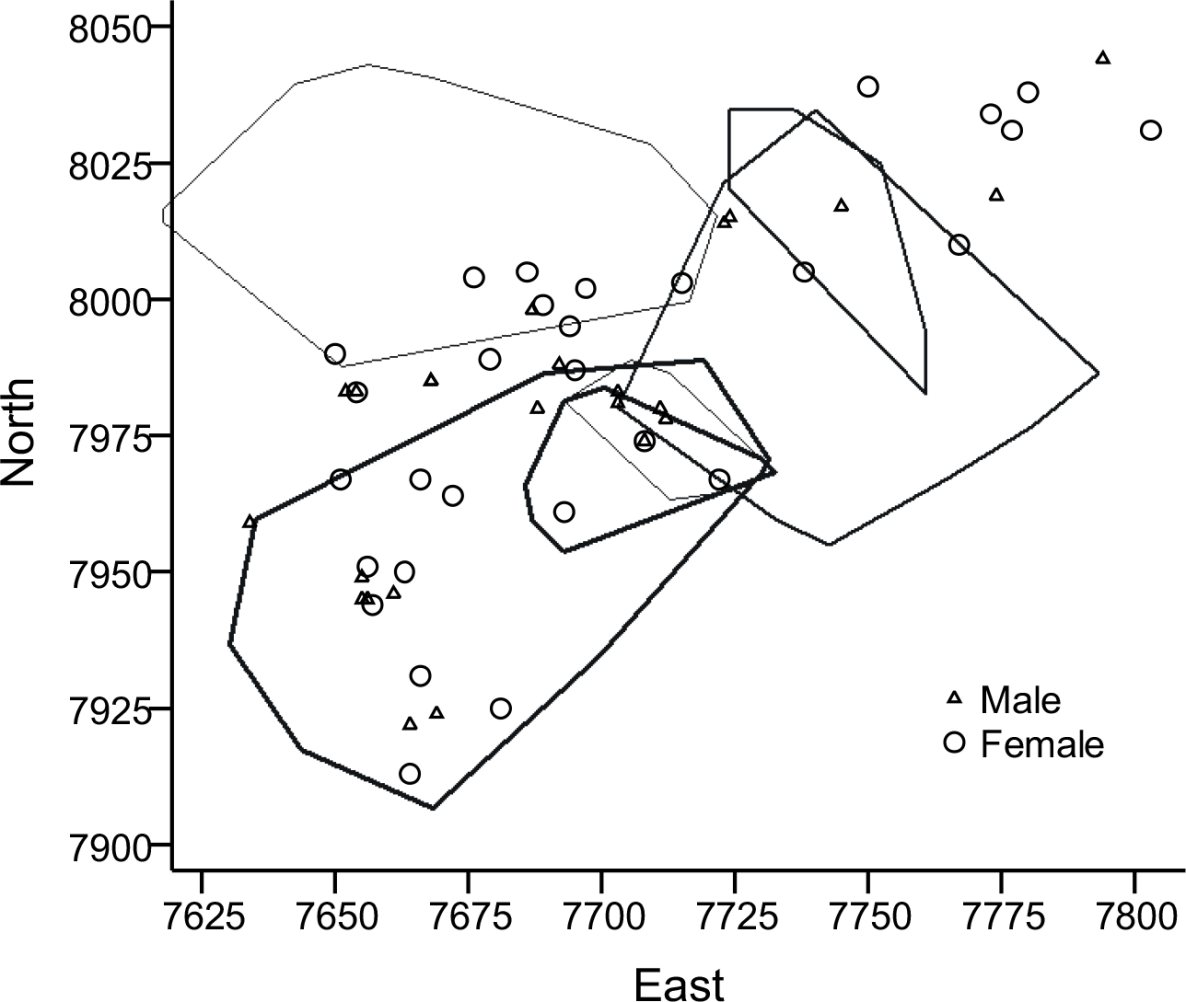
Home ranges of three male water voles in April (subadults) and May (adults) 2009. The three individuals are identified by different line thickness. The distribution of over-wintered males and females represented by the site of first capture is also given (including the three radio collared males). The most north-westerly home range stretches out of the trapped plot. Axes are in meters.

Home range size was affected by sex and age, but not by year (Table 1). Adult males had significantly larger home ranges than adult females (U = 1.0, p*<*0.001, n = 29), on average 3.3 times larger (Table 2), whereas there was no significant difference between those of juvenile males and juvenile females (U = 39.5, p>0.05, n = 19). Juveniles (both sexes combined) had smaller home ranges than adult females (U = 43.0, p = 0.001, n = 34). The average range of adult males was 7 times larger than of juvenile males, adult females had 2.1 times larger ranges than juvenile females. The increase in range by adult males was clearly demonstrated by the three males tracked both in April and May 2009 (Fig 2). On average, these three males increased their home range from 498 to 2805 m between the two sessions, *i*.*e*., between around 17 April and 7 May (Fig 1). Two other males were predated between the two sessions, one of these used an extraordinarily small range, only 51 m in April (not included in home range analyses). At 122 g he was the smallest animal radio-collared (the heaviest was 267 g at the same time) and spent most of his time hiding in the wooden debris of a once small barn, which was visited by many of the radio-collared animals as well as some non-collared voles (some serious chasing was observed there already in April). Including this vole would have reduced mean home range size in juveniles+subadults by 4.4%. The range size of subadults tracked in April were similar to those of juveniles in summer and autumn (2009 vs. 2008: U = 20, p>0.05, n = 7 in both years).

**Table 1:** Factors affecting home range size in water voles.

Source	d.f.	Wald χ^2^	p<
Intercept	1	4469.1	0.001
Age	1	86.9	0.001
Sex	1	60.5	0.001
Year	3	3.4	n.s.

GLMZ using a loglinear Poisson regression model with age, sex and year as factors (Akaike’s information criterion = 8390). Scale parameter method was set to “Deviance”, because of a high degree of variance in the variables. N = 48 voles.

**Table 2:** Home range size (m^2^, 100% minimum convex polygon) of adult and juvenile + subadult water voles in Solvær.

	Mean	SD	N	Min.	Max.	Fixes
Adult ♂♂	2774.0	752.6	14	1446	4134	8069
Adult ♀♀	848.3	394.0	15	328	1581	8789
Juvenile + subadult ♂♂	405.1	145.9	12	239	722	3845
Juvenile + subadult ♀♀	397.0	241.7	7	127	822	2643

SD, standard deviation; N, sample size; Min., minimum; Max., maximum, Fixes, number of fixes.

**Fig 3:**
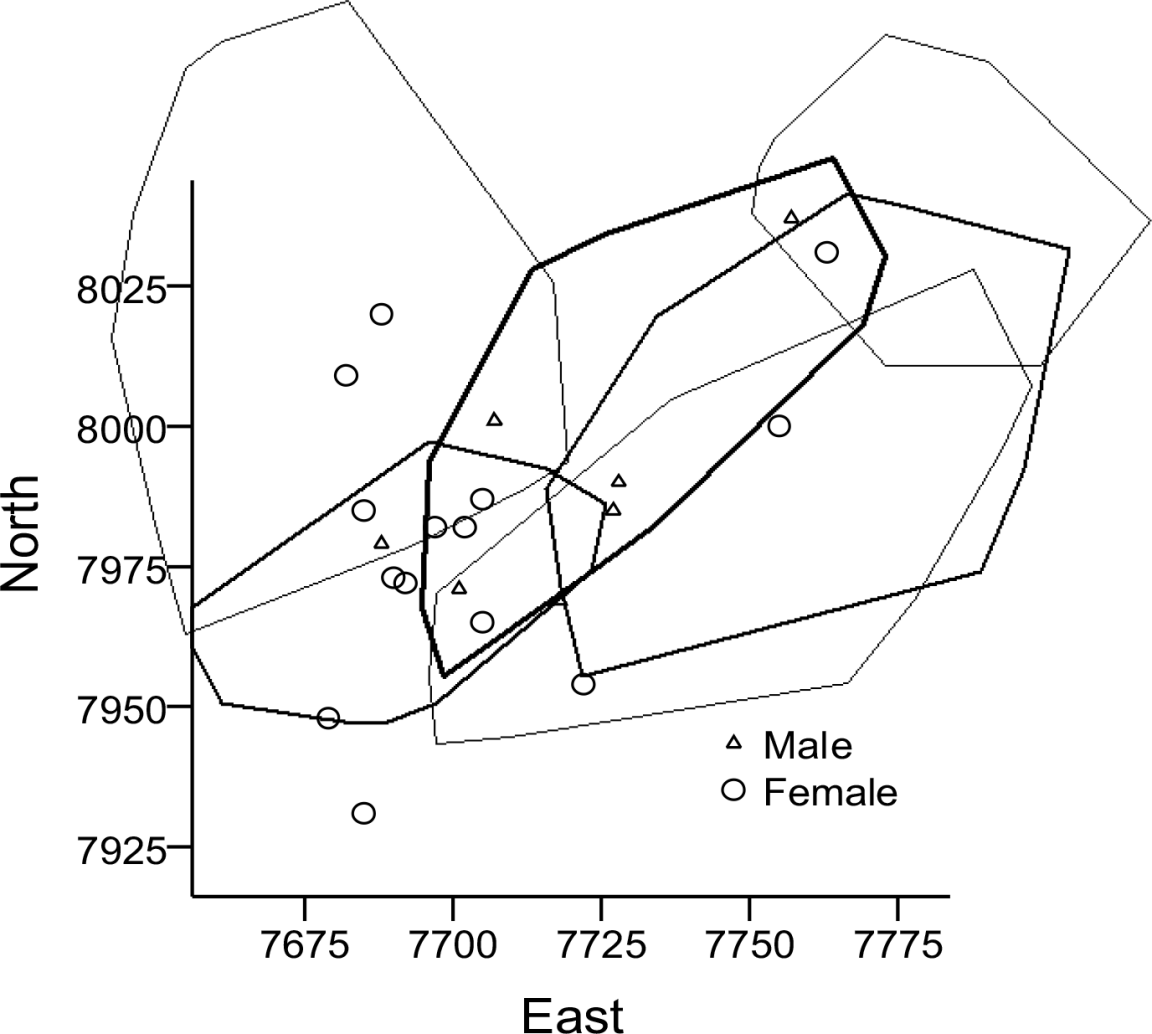
Home ranges of six adult male water voles in 2007. The distribution of adult (over-wintered) males and females represented by the site of first capture is also given (including the six radio collared males). The most north-westerly home range stretches out of the trapped plot. Axes are in meters.

**Fig 4:**
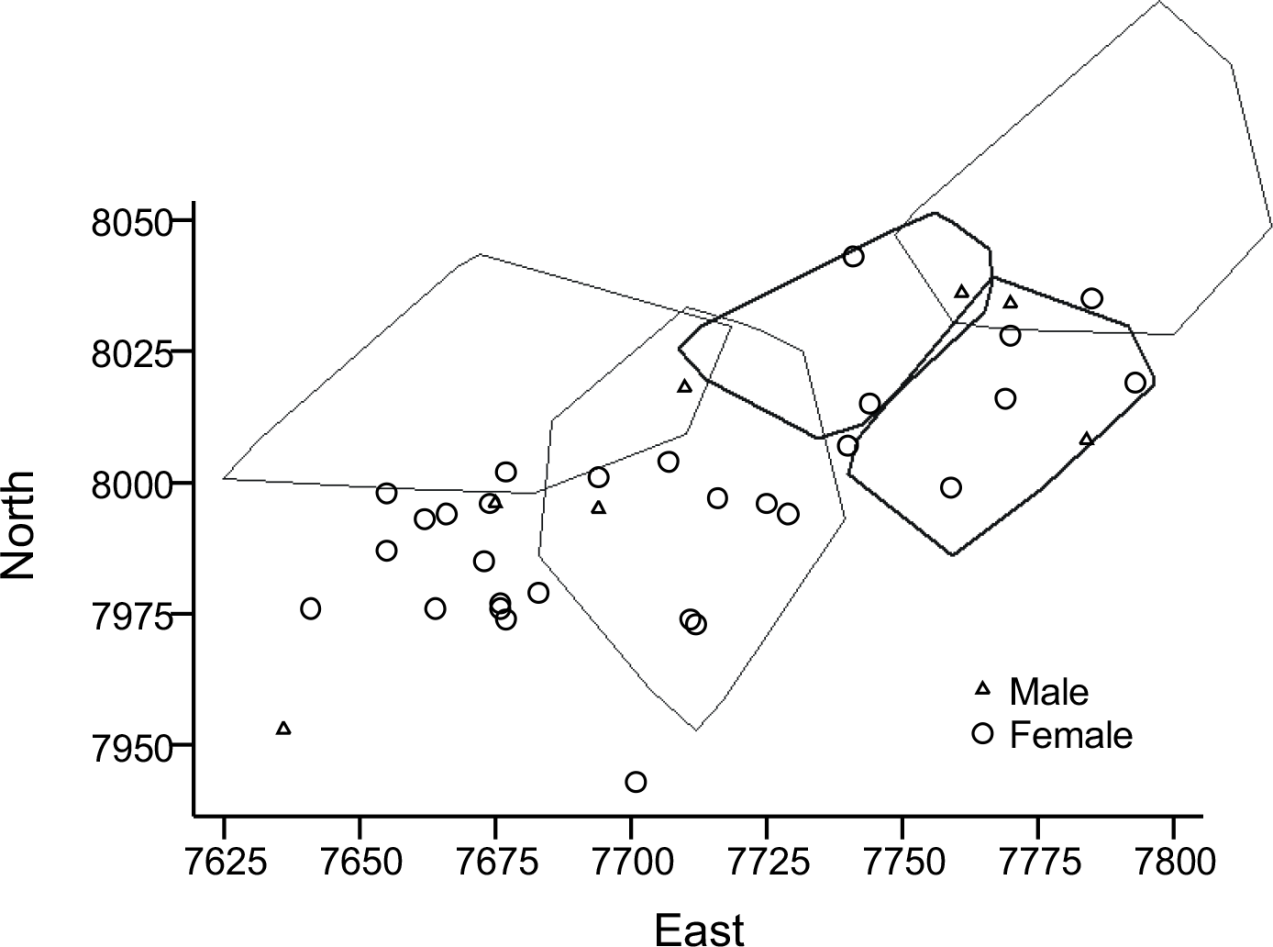
Home ranges of five adult male water voles in 2008. The distribution of adult males and females represented by the site of first capture is also given (including the five radio collared males). The most north-westerly home range stretches out of the trapped plot, the most north-eastern one also slightly. Axes are in meters.

In mid-summer, July and August, the ranges of most males decreased. In 9 males where this could be studied (2007–2008), mean home range size decreased from 2427.9±572.9 m in Session 1 to 1740.9±746.8 m in Session 2 (z = 2.12, p<0.05). Furthermore, in three of these males, mean range in Session 3 dropped further to 1591.3±1049.1 m, with minimum as low as 493 m (*i*.*e*., similar to juvenile range size). In fact, only one of these males increased his range from one session to the next, only to decrease it again by a similar area in the third session. In 8 females (2006–2008), mean home range size was 702.0±231.0 m in Session 1 and 938.3±345.6 m in Session 2 (z = 1.32, p>0.05), and 812.0±458.2 m² in two of these females in Session 3. The number of adults in the study plot varied much between years (Table 3, Figs 2–4).

**Table 3:** Numbers of adult (over-wintered) male and female water voles caught in the study area by year in relation to mass and home range size.

Year	Adult ♂♂	Adult ♀♀	Mass ♂♂	Mass ♀♀	Range ♂♂	Range ♀♀	N
2006	11	21	-	210.0	-	1279.7	3
2007	10^a^	16^a^	228.8	226.5	2503.0	861.7	17
2008	7	33	216.6	225.5	2972.8	731.5	16
2009	26	31	221.7	214.0	2984.7	395.0	8

Adult ♂♂ and ♀♀, total numbers; mass ♂♂ and ♀♀, average body mass in g; range ♂♂ and ♀♀, home range size (m^2^) of radio-collared adult males and females; N, number of radio tracked voles.

^a^Note that the trapped area was smaller in 2007 than in other years.

Average overlap of male home ranges was 488.4±505.0 m, n = 19 overlap zones (interfaces between two ranges), and of females 236.9±258.9 m, n = 13 zones (U = 74, p = 0.06, excluding non-overlapping individuals). Estimated as percentages of average male and female home range, overlap represented 17.0% and 28.8%, respectively. In 2007, home ranges in 4–5 of 6 adult males overlapped much (Fig 3), while 5 males in 2008 showed less overlap (Fig 4); mean = 628.4±597.7 m, n = 11 zones, vs. mean = 348.7±289.0 m, n = 6 zones (U = 22, p>0.05), 21.9% and 12.2% respectively. The ranges of some of the radio-collared juveniles overlapped nearly 100%, presumably being litter mates. Average overlap in juvenile (including subadults) ranges was 135.0±142.0 m² (n = 20 zones), or 33.7%. When also considering adult non-collared animals that lived in the same area (Figs 2–4, Table 3), female-female overlap of ranges must have been extensive and male-male overlap less extensive.

Twenty of the adults and subadults that were radio-collared had been ear-marked as juveniles the previous year (2006–2008). Of these, 16 did not disperse from their natal site, as their home ranges included the first site of capture as juveniles. In 2009, one of the males had non-overlapping ranges in April and May, although separated by merely 10 m (Fig 2). Three more voles also moved their home range slightly (10–15 m) as their adult range did not include the site of capture as juveniles.

**Fig 5:**
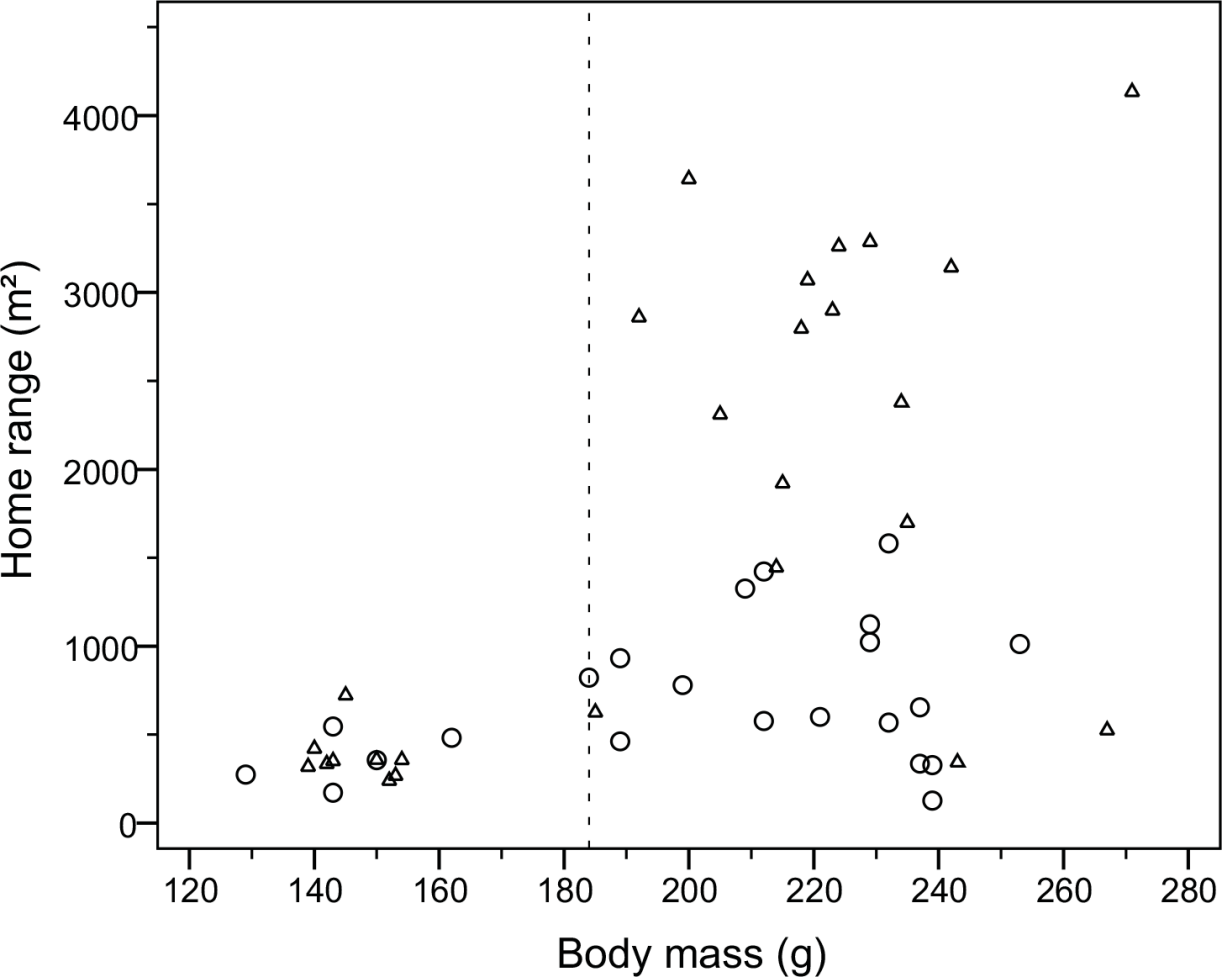
Home range (m^2^) as a function of body mass (g) in female (circles) and male (triangles) water voles. Juveniles are found to the left of the dashed line, adults to the right and subadults on both sides.

Body mass did not influence home range size (Fig 5) in adult males (r = 0.32, p>0.05), adult females (r = -0.04, p>0.05) or juveniles (r = 0.08, p>0.05). The three males in Fig 2 had reached their reproductive mass in April, prior to increasing their range. The heaviest of these males lost 43 g between 9 April and 14 May and attained a mass similar to other males. There was no significant difference in mass between adult males and females (t = 0.23, d.f. = 27, p>0.05), but pregnant females may lead to an overestimation of female mass. Average mass of adults (at first capture) was 222.1±19.0 g (n = 29). The heaviest male (271 g) also lost most mass during the summer (57 g). Among 44 radio-collared water voles, 10 (23%) died between the study sessions. Of these, 8 were predated (most likely by eagle owls) and 2 were found dead *in situ*. In an additional case the transmitter was recovered below the high tide level on a tiny islet 250 m away with no trace of the animal, having probably been predated and air-lifted to the island. Among 29 adults, 11 males and 10 females were still alive when their study period ended, while 3 males and 5 females were dead (test between the sexes: χ = 2.57, d.f. = 1, p>0.05). Home ranges of adult voles that died averaged 86% of the mean for animals still alive (U = 64.0, p>0.05, n = 29, both sexes combined).

## Discussion

The water vole appears to have a social structure similar to that reported in several studies of *Microtus* spp, but without the exclusive female territoriality mandatory for breeding [9, 34–39]. Predictions 1, 3 and 5 were supported, prediction 2 was partly supported, while prediction 4 was unsupported. The size of their home range fits the general relationship of body size to home range among mammals [40],*i*.*e*. Figure 10.4 herein. The shifts in home range size, especially the large increase in adult male ranges at the start of the breeding season, should also have been expected, as well as a reduction in range size of some males at the end of the breeding season [4, 19]. The increase in range was preceded by an increase in body mass, as expected for reproductively active voles. The size of the home ranges also is well within the expected, being at the upper end of range size found in voles relates to the larger size of water voles. For example, in one study of *Microtus oeconomus* males averaged 804 m², with maximum 2756 m² [35], and in a study of the slightly smaller *M*. *agrestis* males averaged *ca*. 1400 m² [8]. For the southern water vole (*Arvicola sapidus*) home ranges up to 2858 m² have been reported [22].

The social structure of water voles was polygamous (promiscuous), as found in many other voles [9, 20, 41]. Dominant males increased their range in the breeding season most likely to maximize (“optimize”) the number of females and consequently their reproductive success (prediction 1 supported). In my study, this did not seem to increase mortality, mainly due to predation, relative to females, but this may have been a side effect of the study. My presence during a large part of the season may have reduced hunting by eagle owls in the study area. Differences in home range size between years could be a proxy for differences in density, but with no such difference being found, prediction 4 was not supported. However, this is likely to vary between habitats and regions [27]. When mating opportunities ceased in July, many males were battle-scared and in poor condition, *sensu* [42].

The much larger ranges of adult males than females (prediction 2 supported) was most likely related to reproduction. If these males were to survive the next winter, they would have to reduce their roaming activity and range size when mating opportunities ceased in late summer. Females did not reduce their range size towards the end of the breeding season, which may be related to the increased number of juveniles and their independence and occupation of parts of the range, *i*.*e*. the density of juveniles. However, because juvenile males did not have larger home ranges than juvenile females, prediction 2 was, perhaps unsurprisingly, not supported for juveniles. Sexual dimorphism in home range size was not associated with dimorphism in body mass, contrary to that found in some voles [34, 35]. This lack of correlation was surprising, but may perhaps be explained by small differences in habitat quality between ranges. More dominant individuals (presumably heavier) may occupy patches of higher quality (or more females) and hence need smaller ranges, although this argument may not be convincing for breeding males, *sensu* [43–45].

As expected, juveniles caught in close proximity, many in identical trap positions, overlapped very much in their home ranges, some overlapped nearly completely. This apparently continued to a large extent throughout the winter until subadults in spring matured reproductively. In most adults, the ranges were simply enlarged with little or no migration out of their natal range. This probably caused the large degree of overlapping ranges found among adult females, in relative terms nearly twice that found among adult males, 29 vs. 17% (although barely significant). The overlap especially for females would have been much larger if the many non-collared voles were also considered. Such overlap in ranges may be caused by a higher tolerance of related than unrelated voles or familiar vs. unfamiliar voles [46]. It appears that congregations in other vole species may include both related and unrelated individuals [4]. None of the radio-collared water voles dispersed long distances, although a few had moved a short distance from their first site of capture as juveniles. This social system can best be described as family (or clan) ranges occupied by extended families, possibly including a female’s subsequent litters, *sensu* [47–51]. Females may best be described as non-territorial, while males may be partly, but not exclusively, territorial (prediction 5 supported).

At the onset of reproduction, females increased their mass as much as males, but their home range size increased much less. Consequently, both females and males adjusted their range size during the season (prediction 3 supported). Females may benefit from mating with several males, but more likely succumbed to forced copulations. No evidence of infanticide was found, hence mating with multiple males as a strategy to counter infanticide seems unlikely, *sensu* [52]. Female clustering as defence against aggressive or infanticidal males [53] also seems unlikely, as does female territoriality against infanticidal females [54]. Although spacing behaviour in small mammals has been studied extensively the ultimate function of territoriality in females is still under debate and little data exists on how territorial behaviour changes during reproductive cycles [55]. Shared ranges may also be a consequence of relaxed competition for food (in a largely grass-eating species) without any specific benefits of group living as such.

## Supporting Information

S1 Table Individual data for all water voles included in the analyses. Home range in m² and weight in g.
(PDF)

S2 Table Overlap in home ranges (m²). Paired overlaps of home ranges in water voles, excluding non-overlapping pairs.
(PDF)
