# Supplementary material for: Influence of Reproductive Status: Home Range Size in Water Voles (Arvicola amphibius)
Source: PLoS One. 2016 Apr 26;11(4):e0154338. doi: 10.1371/journal.pone.0154338 (PMC4846030; doi:10.1371/journal.pone.0154338)
Supplement: S1 Table — Home range in m² and weight in g. (PDF) [file pone.0154338.s001.pdf]

ID	Year	Month(s)	Age	Sex	N tracking days	N sessions	Fixes per session	Overall home range	Session home ranges	Weight	Fate
497	2006	June/July/Sept.	Adult	Female	33	3	539/481/322	1581	1029/1340/1136	232	Alive
491	2006	June/July	Adult	Female	24	2	539/468	932	747/654	189/191	Transmitter removed
484	2006	June/July	Adult	Female	23	2	468/481	1326	953/1326	209	Alive
566	2007	June/July	Adult	Male	32	2	375/367	3142	2653/1708	242	Transmitter lost, alive
545	2007	June/July	Adult	Male	35	2	372/365	3285	2929/858	229/217	Transmitter removed
873	2007	June/July	Adult	Male	33	3	365/366/96	3069	3014/1888	219/213	Transmitter removed
468	2007	June/July	Adult	Male	29	2	362/365/19	2378	2142/1738	234/230	Transmitter removed
586	2007	June/July	Adult	Female	32	3	321/367/92	1012	483/924	253/215	Transmitter removed
366	2007	June/July	Adult	Female	27	2	309/365	654	654/654	237	Predated
906	2007	June-Sept.	Adult	Male	51	4	365/366/350/239	1698	1698/1698/1698	235/208	Transmitter removed
345	2007	July	Adult	Female	14	1	350	1023		229	Predated
324	2007	July	Adult	Female	12	1	349	1124		229	Predated
444	2007	July	Adult	Male	14	1	348	1446		214	Dead in situ
377	2007	July	Adult	Female	14	1	344	577		212	Predated
296	2007	Aug./Sept.	Juvenile	Male	23	2	326/244	239		152/168	Transmitter removed
417	2007	Aug./Sept.	Adult	Female	22	2	296/250	780	780/780	199/198/202	Transmitter removed
437	2007	Aug./Sept.	Juvenile	Female	22	2	282/250	482		162/162	Transmitter removed
324	2007	Aug./Sept.	Juvenile	Male	21	2	279/81	356		154/167/161	Transmitter removed
377	2007	Aug./Sept.	Juvenile	Male	19	2	226/240	722		145/159	Transmitter removed
345	2007	Sept.	Juvenile	Male	9	1	269	268		153/162	Transmitter removed
906	2008	May	Adult	Male	11	1	270	3640		200	Predated
873	2008	May/June	Adult	Male	34	3	261/278/278	4134	2601/3511/2583	271/216/214	Transmitter removed
345	2008	May/June	Adult	Male	23	2	240/297	1921	1846/1309	215/215	Transmitter removed
377	2008	May/June	Adult	Male	35	3	285/298	2859	3188/1162/493	192/181	Transmitter removed
297	2008	May/June	Adult	Male	22	2	279/296	2310	1780/1796	205	Dead in situ
323	2008	May	Adult	Female	12	1	299	568		232/214	Transmitter removed
365	2008	May/June	Adult	Female	23	2	288/298	600	326/504	221/198	Transmitter removed
417	2008	May/June	Adult	Female	30	3	285/298/160	1442	644/1324/488	212/198	Transmitter removed
906	2008	June	Adult	Female	11	1	278	336		237	Predated
437	2008	July/Aug.	Juvenile	Female	17	2	328/80	172		143/148	Transmitter removed
444	2008	July/Aug.	Juvenile	Female	17	2	322/82	546		143/146	Transmitter removed
468	2008	July/Aug.	Juvenile	Female	18	2	328/88	822		184/188	Transmitter removed
586	2008	July/Aug.	Juvenile	Male	14	1	327	358		150/155	Predated
545	2008	July/Aug.	Juvenile	Male	17	2	326/87	335		142/163	Transmitter removed
566	2008	July/Aug.	Juvenile	Male	18	2	327/93	351		143/166	Transmitter removed
525	2008	July/Aug.	Juvenile	Female	17	2	314/89	356		150/148	Transmitter removed
468	2009	April/May	Subadult/adult	Male	17	2	211/110	525/3260		267/224	Transmitter removed
417	2009	April/May	Subadult/adult	Male	19	2	210/237	343/2898		243/223	Transmitter removed
444	2009	April/May	Subadult/adult	Male	20	2	213/280	127/2796		185/218	Transmitter removed
586	2009	April/May	Subadult/adult	Female	20	2	224/268	127/328		155/239	Transmitter removed
604	2009	April	Subadult	Male	9	1	211	318		139	Predated
545	2009	April	Subadult	Male	9	1	209	51		122	Predated, **Exluded**
437	2009	April/May	Subadult	Male	14	1	224	420		140/-	Transmitter removed
525	2009	April/May	Subadult/adult	Female	21	2	224/265	274/462		130/189	Transmitter removed
