# Supplementary material for: Influence of Reproductive Status: Home Range Size in Water Voles (Arvicola amphibius)
Source: PLoS One. 2016 Apr 26;11(4):e0154338. doi: 10.1371/journal.pone.0154338 (PMC4846030; doi:10.1371/journal.pone.0154338)
Supplement: S2 Table — Paired overlaps of home ranges in water voles, excluding non-overlapping pairs. (PDF) [file pone.0154338.s002.pdf]

Year	ID vole A	ID vole B	Overlapp	Sex	Age
2006	497	417	413	Females	Adults
2006	497	484	52	Females	Adults
2007	586	324	170	Females	Adults
2007	366	586	1808	Females	Adults
2007	324	345	918	Females	Adults
2007	377	586	56	Females	Adults
2007	586	345	215	Females	Adults
2007	377	345	354	Females	Adults
2007	377	324	477	Females	Adults
2007	417	345	37	Females	Adults
2007	417	324	71	Females	Adults
2007	566	454	2232	Males	Adults
2007	444	873	406	Males	Adults
2007	468	873	417	Males	Adults
2007	906	566	202	Males	Adults
2007	444	468	548	Males	Adults
2007	545	444	85	Males	Adults
2007	545	468	1059	Males	Adults
2007	566	468	695	Males	Adults
2007	566	444	344	Males	Adults
2007	906	545	682	Males	Adults
2007	906	468	242	Males	Adults
2008	297	345	64	Males	Adults
2008	297	377	252	Males	Adults
2008	377	345	320	Males	Adults
2008	873	345	446	Males	Adults
2008	873	906	871	Males	Adults
2008	906	345	139	Males	Adults
2008	365	323	271	Females	Adults
2008	906	323	1	Females	Adults
2009	417	468	250	Males	Subadults
2009	417	545	2	Males	Subadults
2009	417	604	49	Males	Subadults
2009	437	604	263	Males	Subadults
2009	437	545	37	Males	Subadults
2009	545	604	51	Males	Subadults
2009	468	604	140	Males	Subadults
2009	468	545	4	Males	Subadults
2009	417	444	33	Males	Adults
2009	444	468	242	Males	Adults
2007	377	437	272	_	Juveniles
2008	444	468	460	_	Juveniles
2008	444	545	31	_	Juveniles
2008	468	545	29	_	Juveniles
2008	566	545	307	_	Juveniles
2008	566	468	46	_	Juveniles
2008	566	444	38	_	Juveniles
2008	586	566	314	_	Juveniles
2008	586	545	320	_	Juveniles
2008	586	525	3	_	Juveniles
2008	586	468	42	_	Juveniles
2008	586	444	41	_	Juveniles
